# Is There Any Relationship between Plasma IL-6 and TNF-*α* Levels and Lumbar Disc Degeneration? A Retrospective Single-Center Study

**DOI:** 10.1155/2022/6842130

**Published:** 2022-01-19

**Authors:** Youfeng Guo, Chao Li, Beiduo Shen, Ziqi Zhu, Xianzhen Chen, Tao Hu, Desheng Wu

**Affiliations:** ^1^Department of Spine Surgery, Shanghai East Hosipital, School of Medicine, Tongji University, Shanghai 200092, China; ^2^Department of Neurosurgery, Shanghai Tenth People's Hospital, School of Medicine, Tongji University, Shanghai 200092, China

## Abstract

Intervertebral disc degeneration (IDD) is one of the most common degenerative diseases all over the world. A growing number of studies have proved that large amounts of cytokines are produced during the development of IDD, and the inflammatory responses induced by these cytokines aggravate the occurrence and development of the disc degeneration. In this retrospective single-center study, a total of 182 lumbar spine cases were retrospectively reviewed between July 2020 and October 2021. An appropriate cutoff value was found for discriminating severity of IDD by William rank-sum test and locally weighted scatterplot smoothing algorithm. The cumulative grade was also calculated by summing Pfirrmann grades for all lumbar spine intervertebral discs. It was found that high-score group (total score > 18) plasma interleukin-6 (IL-6) concentration was significantly higher than that of the low-score group (total score ≤ 18) (9.6 ± 1.75 vs. 5.40 ± 0.61 pg/ml, *p* = 0.002), tumor necrosis factor-*α* (TNF-*α*) following the same trend (5.27 ± 1.48 vs. 2.97 ± 0.23, *p* = 0.006), which was most pronounced in the upper lumbar intervertebral discs (L1-3). In the entire sample, preoperative IL-6 concentration was significantly higher than that of the postoperation (*p* < 0.001), while the TNF-*α* was the opposite (*p* = 0.039). It was also found that there were significant differences in the two groups with respect to age and hypertension (*p* < 0.001 and *p* = 0.037). In conclusion, this study preliminarily indicated the relationship between IL-6 and TNF-*α* and the severity of lumbar disc degeneration.

## 1. Introduction

Intervertebral disc degeneration (IDD) is a significant worldwide health concern, causing 26 to 42% chronic low back pain [1, 2] and imposing a huge economic burden on society [3]. The mechanisms for the development and progression of IDD are extremely complicated. Collectively, the lumbar intervertebral disc degeneration is strongly linked to cellular senescence, stromal metabolism disorders, and enhanced inflammatory response [4–7]. A number of clinical studies have indicated that the activation of the immune response facilitated the development of inflammation [8, 9]. Cytokines play an important role in inflammation response [10]. And the inflammatory response, in turn, also elevates the circulating levels of degeneration-promoting inflammatory cytokines such as c-reactive protein (CRP) and interleukin-6 (IL-6). Many molecular biology studies [11–13] confirmed that the expression of inflammatory factors in the degenerative intervertebral disc was increased. Further studies confirmed that IL-6, interleukin-1*β* (IL-1*β*), and interleukin-10 (IL-10) could mediate inflammatory response and accelerate the degeneration of lumbar intervertebral disc [14, 15]. Simultaneously, it has also been suggested that elevated plasma concentrations of cytokines were not only associated with degree of IDD but also severity of low back pain [16].

However, studies on the clinical significance of preoperative and postoperative plasma levels of cytokines are rare in the existing literature, which only compared the postoperative infection with inflammatory indicators such as CRP, and did not compare longitudinal changes between pre- and postoperative data [17, 18]. Here, we evaluated the preoperative value of plasma cytokines for the degree of intervertebral disc degeneration and evaluation value of surgical prognosis, aimed at enhancing in-depth understanding of cytokines and giving spine surgeons a guide for the preoperative and postoperative assessment of these indexes, given that cytokines correlate with IDD.

## 2. Methods

### 2.1. Participant Population

The study was conducted at the department of Spine Surgery, in Shanghai East Hospital. In this study, 182 patients with low back pain as the chief complaint were enrolled between July 2020 and October 2021. The sampling procedure began at the same time. The study was approved by the hospital ethics committee, and written informed consent for publication was obtained from the patient.

### 2.2. Inclusion and Exclusion Criteria

Inclusion criteria are as follows: (1) symptoms: low back pain with unilateral or bilateral lower limb nerve root radiation pain; (2) special nerve root stimulation test: straight leg improvement test, strengthening test, dorsal extension test, or femoral nerve tension test positive; (3) numbness and fatigue in one lower limb or both lower limbs or lack of corresponding knee reflex or ankle reflex; and (4) CT or magnetic resonance imaging (MRI) examination confirmed signs of disc herniation (defined as pressure on and stimulation of the spinal marrow or nerve root) or spinal canal stenosis (defined as a spinal canal mid-sagittal diameter of <12 mm [19]).

Exclusion criteria are as follows: (1) combined with spinal cord tumor, (2) history of spinal cord trauma, (3) combined with intervertebral space infection, (4) the corresponding segment of the intervertebral disc has a history of previous surgery or lumbar vertebral compression fracture, and (5) known inflammatory condition (e.g., osteomyelitis, systemic lupus erythematosus, rheumatoid arthritis (RA), and polymyositis).

### 2.3. Clinical Data Collection, Blood Sample Management, and Evaluation Criteria

We collected peripheral blood samples twice for biomarker measurements. All blood samples were collected between 6.00 am and 9.00 am the day after admission under fasting conditions. In addition, gender, age, height, weight, and BMI as well as diabetic mellitus (DM), hypertension, cerebral infarction, and Parkinson's disease history were assessed in this study. The patients were operated via a standard posterior surgical decompression approach. All the resected intervertebral discs were responsible intervertebral discs confirmed with intraoperative fluoroscopy prior to removal. Blood collection protocol twelve-hour fasting blood samples were collected again between 6.00 am and 9.00 am on the third postoperative day. Vein blood was collected from the patients and then placed in the anticoagulated tubes with EDTA. The blood samples would be sent for centrifugation at 3000 rpm and 5 minutes as soon as possible. The plasma samples were obtained through aspirating the supernatant carefully, and all the plasma samples were kept at −80°C until use.

The Pfirrmann grade (1-5) [20] was used to evaluate the severity of IDD by magnetic resonance images, read by two experienced spine surgeons who analyzed each subject in a blind fashion. The cumulative grade was also calculated by summing Pfirrmann grades for all lumbar spine intervertebral discs. Discrepant results will be resolved through negotiation and, if necessary, consultation with the third experienced spine surgeon.

### 2.4. Determination of Cytokines and Proinflammatory Factors

The biochemist (NG) performing the laboratory studies was blinded about the patients' clinical and radiological findings. Biochemical preparation and analysis of the samples were conducted according to the manufacturer's protocols. IL-6 and TNF-*α* levels in the plasma were measured using IL-6 and TNF-*α* ELISA kit (Saiji Bio, China), which is commercially available.

### 2.5. Statistical Analysis

Data were presented as mean values with standard error of the mean (SEM), numbers, or percentages. Category variables were compared with Pearson Chi-square test or Fisher's exact test. Continuous variables were compared between two groups with unpaired Student's *t*-test or William rank-sum test where appropriate. The linear correlation between two continuous data was detected with the Pearson linear analyses. To find the cutoff value of the cumulative grade, William rank-sum test was also performed with IL-6 and TNF-*α* grouped by each score of cumulative grade, and the *p* values were adjusted by locally weighted scatterplot smoothing algorithm. The level of significance was set at *p* < 0.05, two tailed. Collected data were encoded into R software (Version 4.0.5) and SPSS 25.0 and analyzed.

## 3. Results

### 3.1. Participant Characteristics

The demographic characteristics of enrolled patients are described in Table 1. After excluding inappropriate participants, a total of 182 IDD patients were recruited in this study. The mean age was 63.5 ± 0.9 years old, and the BMI was 24.5 ± 0.3 kg/m^2^. Female patients had a relatively large proportion (54.4%) and 45.6% were male. For the comorbidities, the most common disease was hypertension (45.1%), followed by diabetes (14.3%) and coronary heart disease (11.0%).

The Pfirrmann grading system revealed the severity of disc degeneration from L1/2 to L5/S1 (Table 2). For L1/2, most patients (49.5%) were graded 2. And for L2/3, the distribution of subjects was relatively uniform. Patients in grade 2, 3, 4, and 5 were 48 (26.4%), 50 (27.5%), 37 (20.3%), and 47 (25.8%), respectively. Patients grading 3 accounted for the largest in L3/4 (31.3%). Most patients were in grade 5 for L4/5 (39.6%) and L5/S1 (54.4%).

As for the cumulative grade, the lowest was 9 and the highest was 25. There were 31 (17.0%) patients graded between 9 and 14, 71 (39.0%) patients graded between 15 and 18, and 50 (27.5%) patients graded between 19 and 22. Most patients were in score 17 (12.1%).

### 3.2. Severity Classification

**Figure 1:**
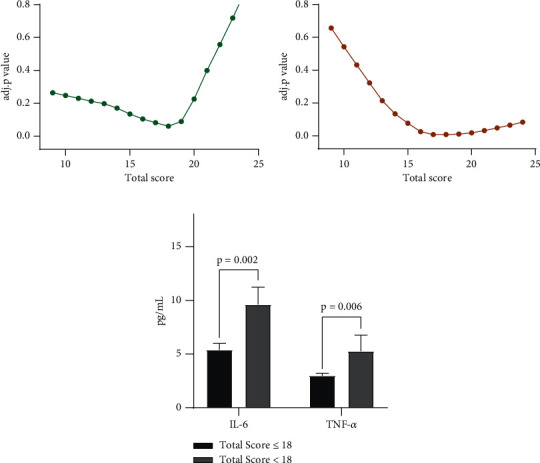
LOWESS curve was performed for searching cutoff value for IL-6 (a) and TNF-*α* (b). Comparison of IL-6 and TNF-*α* concentrations in the groups of low score (total score ≤ 18) and high score (total score > 18) (c).

The mean plasma IL-6 and TNF-*α* level was 7.24 ± 0.89 pg/ml and 3.98 ± 0.66 pg/ml, respectively (Table 3). Correlation analysis showed that both of them were related to the cumulative grade (IL-6, *r* = 0.176, *p* = 0.018; TNF-*α*, *r* = 0.241, *p* = 0.001). Besides, mean concentrations of IL-1*β*, IL-2, IL-8, and IFN-*γ* were 4.36 ± 0.66 pg/ml, 2.74 ± 0.12 pg/ml, 33.49 ± 2.00 pg/ml, and 2.87 ± 0.10 pg/ml, respectively. Preliminary analysis also revealed no correlation between cumulative grade and IL-1*β*, IL-2, IL8, and IFN-*γ* (all *p* > 0.05) (Table 3). The *p* value of the Mann–Whitney *U* test adjusted by LOWESS for IL-6 and TNF-*α* are shown in Figures 1(a) and 1(b). Both of the two curves showed that the cutoff value of cumulative grade to discriminate severity of disc degeneration was 18. And plasma levels of IL-6 and TNF-*α* were significantly different between the low- and high-score groups (IL-6, 5.40 ± 0.61 pg/ml for the low-score group vs. 9.6 ± 1.75 for the high-score group, *p* = 0.002; TNF-*α*, 2.97 ± 0.23 pg/ml vs. 5.27 ± 1.48, *p* = 0.006; Figure 1(c)).

In the entire sample, 102 (56.0%) and 80 (44.0%) of the participants were determined to the low-score group (total score ≤ 18) and high-score group (total score > 18), respectively. There was no statistical difference between these two patient groups in terms of BMI (*p* = 0.231). The proportion of female patients was marginally greater in the high-score group (*p* = 0.052). A statistically significant difference was observed between groups with respect to age (*p* < 0.001). For the history of previous diseases, there was no statistically significant difference with coronary heart disease, diabetes, cerebral Infarction, and Parkinson's disease (*p* = 0.921, 0.807, 0.474, and 0.782, respectively). Notably, a statistically significant difference was observed in hypertension (*p* = 0.037).

**Figure 2:**
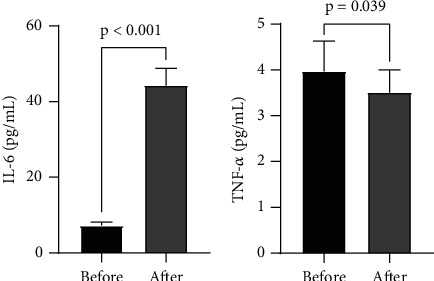
Comparison of IL-6 (a) and TNF-*α* (b) concentrations before and after surgery.

The presurgery and postsurgery plasma concentrations of IL-6 and TNF-*α* are shown in Figure 2. As indicated by our data (Figure 2(a)), the plasma concentration of IL-6 at postsurgery was significantly higher than that of presurgery (*p* < 0.001). In sharp contrast, the plasma TNF-*α* concentration showed a significant reduction after surgery (*p* = 0.039; Figure 2(b)). There was also a statistical difference in postsurgical plasma IL-6 levels between the low- and high-score groups (*p* = 0.034).

### 3.3. Analysis in Different Lumbar Intervertebral Discs

Table 2 shows the Pfirrmann grade of each disc. For L1/2 and L2/3, most patients (72.5% and 46.1%, respectively) are grade 2 in the low-score group, followed by grade 3 (20.6% and 38.2%, respectively), while the majority of participants (32.5% and 55.0%) was grade 5 in the high-score group. In terms of L3/4, L4/5, and L5/S1, the number of patients occupying the largest proportion was, respectively, grade 3 (49.0%), 4 (44.1%), and 5 (35.5%) in the low-score group. In striking contrast, the percentage of patients who are grade 5 was consistently the biggest (62.5%, 70.0%, and 78.8%) in the high-score group with L3/4, L4/5, and L5/S1, respectively.

**Figure 3:**
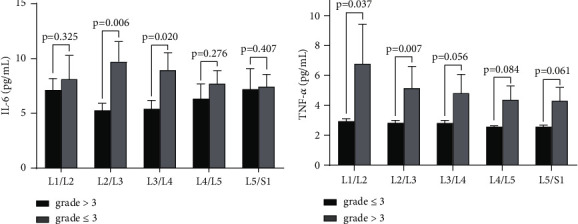
(a) Comparison of IL-6 concentrations in the groups of mild to moderate degeneration (grade ≤ 3) and severe degeneration (grade > 3) in terms of a single disc. (b) Comparison of TNF-*α* concentrations in the groups of mild to moderate degeneration (grade ≤ 3) and severe degeneration (grade > 3) in terms of a single disc.

We classified 1, 2, and 3 in the Pfirrmann score system as the mild to moderate degeneration group and 4 and 5 as the severe degeneration group for a single-disc segment. As illustrated in Figure 3(a), from L1/2 to L5/S1, the mean plasma concentration of IL-6 was substantially higher in the severe degeneration group compared with the mild to moderate degeneration group. Among these, differences in L2/3 and L3/4 between two groups were found to be statistically significant (*p* = 0.006 and 0.020, respectively). The other three disc comparisons are not statistically significant (all *p* > 0.05). Similar to IL-6, serum TNF-*α* concentration in the severe degeneration group was also significantly higher than the mild to moderate degeneration group. There were statistically significant differences in TNF-*α* concentration in L1/2 and L2/3 (*p* = 0.037 and 0.007, respectively; Figure 3(b)), and a borderline statistical significance was observed in L3/4 (*p* = 0.056). No statistically significant correlations were observed in the other two discs (*p* = 0.084 in L4/L5 and 0.061 in L5/S1).

## 4. Discussion

In this study, we investigated the potential association between the levels of cytokines and the severity of lumbar disc degeneration. Patients were divided into the low- and high-score groups by the cumulative grade of intervertebral disc degeneration, and the cut-off value was 18 according to the levels of IL-6 and TNF-*α*. The difference of the Pfirrmann grade for each disc segment between two groups further demonstrated the successful grouping according to this cut-off value. The differences of IL-6 and TNF-*α* also remained in the analysis of a single-disc segment.

Interleukin is a cytokine superfamily, which can be synthesized and secreted by most cells in vivo and then have effects on a variety of cells. It is considered extremely significant in the activation of immune cells, production of immune active substances, and regulation of immune response. IL-6, an important mediator of immune responses and inflammation in the interleukin family, also plays an important role in the process of lumbar disc degeneration [21]. Suzuki et al. found that IL-6 detected in the degenerative lumbar disc tissue stimulated the aggregation and activation of inflammatory cells and the release of inflammatory transmitters, inducing the autoimmune response of the intervertebral disc, which suggested that IL-6 is highly critical in inflammation during lumbar disc degeneration [11]. Previous studies also have shown that IL-6 was significantly increased in intervertebral disc degeneration tissues and positively correlated with the degree of intervertebral disc degeneration and symptom severity. For example, Du et al. [22] found that serum IL-6 levels were elevated during disc degeneration, and the concentration could predict the severity of symptoms. Our results also showed a statistically significant difference in serum IL-6 levels between the high-score group (total score > 18) and the low-score group (total score ≤ 18). Our results also revealed that from L1/2 to L5/S1, participants with severe degeneration (score > 3) had higher serum IL-6 concentration than those with mild to moderate degeneration (score ≤ 3) in terms of a single disc. These differences were significant in the upper lumbar region (L2/3 and L3/4) and not in the lower lumbar region (L4/5 and L5/S1). Plasma IL-6 concentrations were statistically different between preoperation and postoperation (Figure 2(a)). A probable cause for this observation may be surgical stimulus resulting in increased IL-6 levels. TNF-*α* is a kind of pleiotropic proinflammatory cytokines, deeply involved in a variety of pathological processes of intervertebral disc degeneration, including inflammatory responses and apoptosis. It has been shown that TNF-*α* might be a driver of intervertebral disc degeneration [23]. Lai et al. [16] found TNF-*α* was involved in promoting the progression of IDD and inducing pain exacerbation. The results from these studies are generally in concordance with our findings. Our study found a statistically significant difference in serum TNF-*α* levels between the high-score group (total score > 18) and the low-score group (total score ≤ 18) for the total score of the lumbar disc degeneration scoring system (Figure 1(c)). With respect to a single disc, participants with severe degeneration (score > 3) had significantly higher serum IL-6 concentration than those with mild to moderate degeneration (score ≤ 3) in L1/2 and L2/3 (Figure 3(b)). Interestingly, as opposed to IL-6, we observed that plasma TNF-*α* level of preoperation was statistically higher than postoperation. This also indicated that TNF-*α* might be an important driver during IDD, leading to changes as mentioned above regardless of surgery stimulation. And in this study, we observed inconsistent trends in IL-6 and TNF-*α* after surgery, which may indicate that TNF-*α* plays a more critical role in intervertebral disc degeneration. In addition, despite the presence of surgical stimulation, TNF-*α* still showed the changes described above, suggesting that TNF-*α* may be closely related to intervertebral disc degeneration and less affected by external factors, which can be used as a relatively robust prognostic indicator and applied in clinical practice.

The current mainstream view revealed that the severity of IDD continuously increased with age, reflecting the natural progression of disc degeneration in vivo [24, 25]. The results of the present study suggested that age, as an important risk factor for IDD, was considered to correlate positively with the degree of disc degeneration, consistent with these previous studies [26]. It should be pointed out that numerous studies have shown that excluding interference from other inflammatory diseases, the expression of IL-6 and TNF-*α* was associated with aging, and the mechanisms were not well defined [27, 28]. The relationship between obesity and the degree of intervertebral disc degeneration with BMI as the main evaluation standard has always been a research hotspot. However, the mechanism of the impact of BMI on the severity of IDD is not clear and scholars believe that there are several possible mechanisms, including biomechanical [29] and inflammatory factors [30]. Among the IDD risk factors that we investigated, high BMI was not significantly associated with severity of IDD. The likely reason for this might be our small sample size.

When analyzing some chronic diseases histories of participants, we did not find a statistically significant association between IDD and coronary heart disease, diabetes, cerebral Infarction, and Parkinson's disease (all *p* > 0.05). Interestingly, statistically significant differences between the high- and low-score groups were indicated with respect to hypertension. One possible explanation was that the average age of the high-score group was greater than that of the other group, and age was an obvious risk factor of both IDD and hypertension. And there have been several studies on the effect of hypertension on IDD [31, 32]. For example, Sun et al. [31] found that the tissue renin-angiotensin system, a potential mechanism of developing hypertension, might contribute to IDD by oxidative stress and inflammatory reaction.

There are several notable merits and limitations on this study. Firstly, previous researches mainly focused on the relationship between plasma cytokine levels of preoperative or nonsurgical patients and severity of intervertebral disc degeneration, but our study not only included the preoperative levels but also compared the preoperative and postoperative cytokine levels. Beyond this, the relationship between cytokines and intervertebral disc degeneration was comprehensively analyzed with individual and cumulative scores of the Pfirrmann grade. Of course, we acknowledge that due to our relatively small sample size, the effect of some risk factors on disc degeneration might not be well manifested. Moreover, although the Pfirrmann grade is widely used in classifying disc degeneration, it has the limitation of morphological analysis less intuitive than histology. Apart from that, blood samples for a long time after surgery could not be obtained, which greatly affected our experimental results by surgical stimulation. In addition, the postoperative blood collection time is also worth further in-depth discussion, because intraoperative use of antibiotics or pills may affect the results. This point demands further experimental confirmation.

## 5. Conclusion

In summary, plasma IL-6 and TNF-*α* concentrations were positively correlated with the degree of lumbar disc degeneration, and this association was most pronounced in the upper lumbar discs. The 18 might be a good cutoff value of the cumulative score of the Pfirrmann grade for discriminating low and high severity patients. Age and hypertension may also correlate significantly with disc degeneration. And IL-6 increased after surgery while TNF-*α* decreased. In the future, we will extend long-term follow-up and expand the sample size to determine the relationship between cytokines and IDD severity.

## Figures and Tables

**Table 1:** Demographic characteristics of patients with disc degeneration disease.

	All	Low-score group	High-score group	*p* value
Subjects, *n* (%)	182	102 (56.0)	80 (44.0)	
Age (years)	63.5 ± 0.9	59.9 ± 1.4	68.1 ± 0.9	**<**0.001
Gender				0.052
Male, *n* (%)	83 (45.6)	53 (52.0)	30 (37.5)	
Female, *n* (%)	99 (54.4)	49 (48.0)	50 (62.5)	
BMI (kg/m^2^)	24.5 ± 0.3	24.0 ± 0.5	25.0 ± 0.4	0.231
IL-6 (pg/ml)	7.24 ± 0.89	5.40 ± 0.61	9.6 ± 1.75	0.002
TNF-*α* (pg/ml)	3.98 ± 0.66	2.97 ± 0.23	5.27 ± 1.48	0.006
Other diseases				
Hypertension	82 (45.1)	39 (38.2)	43 (53.8)	0.037
Coronary heart disease	20 (11.0)	11 (10.8)	9 (11.3)	0.921
Diabetic mellitus	26 (14.3)	14 (13.7)	12 (15.0)	0.807
Cerebral infarction	8 (4.4)	3 (2.9)	5 (6.3)	0.474
Parkinson	5 (2.7)	2 (2.0)	3 (3.8)	0.782

Values are expressed as *n* (%) or mean ± SEM. BMI: body mass index; IL-6: interleukin-6; TNF-*α*: tumor necrosis factor-*α*.

**Table 2:** The Pfirrmann grading system for lumbar disc degeneration.

	1	2	3	4	5
All (*n* = 182)					
L1/2	0	90 (49.5)	43 (23.6)	22 (12.1)	27 (14.8)
L2/3	0	48 (26.4)	50 (27.5)	37 (20.3)	47 (25.8)
L3/4	2 (1.1)	24 (13.2)	57 (31.3)	46 (25.3)	53 (29.1)
L4/5	2 (1.1)	8 (4.4)	35 (19.2)	65 (35.7)	72 (39.6)
L5/S1	0	10 (5.5)	30 (16.5)	43 (23.6)	99 (54.4)
Low-score group (*n* = 102)					
L1/2	0	74 (72.5)	21 (20.6)	6 (5.9)	1 (1.0)
L2/3	0	47 (46.1)	39 (38.2)	13 (12.7)	3 (2.9)
L3/4	2 (2.0)	24 (23.5)	50 (49.0)	23 (22.5)	3 (2.9)
L4/5	2 (2.0)	8 (7.8)	31 (30.4)	45 (44.1)	16 (15.7)
L5/S1	0	10 (9.8)	25 (24.5)	31 (30.4)	36 (35.3)
High-score group (*n* = 80)					
L1/2	0	16 (20.0)	22 (27.5)	16 (20.0)	26 (32.5)
L2/3	0	1 (1.3)	11 (13.8)	24 (30.0)	44 (55.0)
L3/4	0	0	7 (8.8)	23 (28.7)	50 (62.5)
L4/5	0	0	4 (5.0)	20 (25.0)	56 (70.0)
L5/S1	0	0	5 (6.3)	12 (15.0)	63 (78.8)

Values are expressed as *n* (%).

**Table 3:** Correlation test for six cytokines.

	Mean ± SEM	*r*	*p* value
IL-1*β*	4.36 ± 0.66	0.094	0.208
IL-2	2.74 ± 0.12	0.137	0.067
IL-6	7.24 ± 0.89	0.176	0.018
IL-8	33.49 ± 2.00	0.138	0.064
IFN-*γ*	2.87 ± 0.10	-0.031	0.681
TNF-*α*	3.98 ± 0.66	0.241	0.001

Values are expressed as mean ± SEM. IL: interleukin; IFN-*γ*: interferon gamma; TNF-*α*: tumor necrosis factor-*α*.

## Data Availability

The datasets generated and/or analyzed during the present study are available from the corresponding author upon reasonable request.
